# CAG Postdoctoral - A1HELMINTH-INDUCED IL-33 PROMOTES TISSUE-REPAIR MACROPHAGES THAT PROTECT AGAINST COLITIS INDEPENDENT OF IL-4 SIGNALING

**DOI:** 10.1093/jcag/gwaf042.001

**Published:** 2026-02-13

**Authors:** L Kraemer, P Volk, A Wang, H McSorley, D McKay

**Affiliations:** University of Calgary, Calgary, AB, Canada; University of Calgary, Calgary, AB, Canada; University of Calgary, Calgary, AB, Canada; University of Dundee, Dundee, Scotland, United Kingdom; University of Calgary, Calgary, AB, Canada

## Abstract

**Background:**

Infection with helminth parasites induces alarmin release, including IL-33 that initiates type-2 immunity. In non-permissive hosts, helminth expulsion is often critically dependent on IL-4Rα–dependent signaling; indeed, IL-4Ra^-/-^ mice fail to expel the tapeworm *Hymenolepis diminuta*, while increased IL-33 in the gut indicates that the IL-4Rα^-/-^ mouse is ‘aware’ of the worm’s presence. Surprisingly, *H. diminuta*-infected IL-4Rα^-/-^ mice experience less severe DNBS-colitis. Thus, we hypothesised that IL-33 can play a role in mediating the helminth-induced anti-colitic effect.

**Aims:**

To determine whether IL-33 plays a protective role in *H. diminuta* infection-induced suppression of colitis.

**Methods:**

BALB/c WT and IL-4Rα^-/-^ mice were orally infected with five *H. diminuta*. At eight days post-infection, colitis was induced with DNBS (2.5mg, i.r.) and disease assessed 72h later. Colon tissue was analyzed by qPCR, ELISA and spectral flow cytometry. Some mice were treated with anti-CSF1R (200μg) to deplete macrophages or with HpBARI_Hom2 (10μg), a protein blocker of IL-33/ST2 signaling. To test the direct effect of IL-33 on macrophages, bone marrow-derived macrophages were treated with IL-33 (20ng/mL, 120h) and assessed for mannose receptor (CD206), arginase-1 and IL-10 expression by qPCR and flow cytometry. The impact of i.p.-delivered IL-33 treated macrophages (M(IL33); 10^6^ cells) on DNBS-induced colitis was examined.

**Results:**

IL-33 mRNA and protein were significantly increased in the small intestine and colon of *H. diminuta*-infected mice, and this was accompanied by increased numbers of CD206+ST2+ colonic macrophages. Blocking IL-33/ST2 signaling or depleting macrophages abolished the protective effect evoked by *H. diminuta* against DNBS-induced colitis in WT and IL-4Rα^-/-^ mice, indicating that the mechanism is independent of canonical type-2 immune signaling. *In vitro*, IL-33 evoked up-regulation of CD206 and arginase-1, suggestive of a repair/regulatory macrophage. Adoptive transfer of M(IL33)^WT^ or M(IL33)^IL-4Rα-/-^ provided substantial protection against DNBS-induced colitis, confirming the cells regulatory capacity. The finding with M(IL33) from IL-4Rα^-/-^ mice highlights that the anti-colitic effect is not dependent on IL-4Rα signaling on macrophages.

**Conclusions:**

Infection with *H. diminuta* induces increased colonic IL-33 production which promotes the generation of regulatory macrophages—M(IL-33)—capable of exerting anti-inflammatory effects via an IL-4Rα-independent mechanism. These findings reveal a novel IL-33–macrophage axis as a key component of helminth-elicited suppression of concomitant disease and could represent a new therapeutic approach to IBD.

**Funding Agencies:**

CAG, CCC, CIHR

